# A Specific Diplotype H1j/H2 of the MAPT Gene Could Be Responsible for Parkinson's Disease with Dementia

**DOI:** 10.1155/2020/8813344

**Published:** 2020-12-03

**Authors:** Imane Smaili, Imane Hajjaj, Rachid Razine, Houyam Tibar, Ayyoub Salmi, Naima Bouslam, Ahmed Moussa, Wafa Regragui, Ahmed Bouhouche

**Affiliations:** ^1^Research Team in Neurology and Neurogenetics, Genomics Center of Human Pathologies, Faculty of Medicine and Pharmacy, University Mohammed V, Rabat, Morocco; ^2^Department of Neurology, Hassan II Regional Hospital Center, Agadir, Morocco; ^3^Laboratory of Public Health, Faculty of Medicine and Pharmacy, University Mohammed V, Rabat, Morocco; ^4^Department of Neurology and Neurogenetics, Specialities Hospital, Rabat, Morocco; ^5^Laboratory of Information and Communication Technologies, National School of Applied Sciences, Abdelmalek Essaadi University, Tanger, Morocco

## Abstract

Parkinson's disease (PD) is the second most common neurodegenerative disorder after Alzheimer disease. Five to ten percent of patients have monogenic form of the disease, while most of sporadic PD cases are caused by the combination of genetic and environmental factors. Microtubule-associated protein tau (MAPT) has been appointed as one of the most important risk factors for several neurodegenerative diseases including PD. MAPT is characterized by an inversion in chromosome 17 resulting in two distinct haplotypes H1 and H2. Studies described a significant association of MAPT H1j subhaplotype with PD risk, while H2 haplotype was associated with Parkinsonism, particularly to its bradykinetic component. We report here an isolated case displaying an akinetic-rigid form of PD, with age of onset of 41 years and a good response to levodopa, who developed dementia gradually during the seven years of disease progression. The patient does not carry the LRRK2 G2019S mutation, copy number variations, nor pathogenic and rare variants in known genes associated with PD. MAPT subhaplotype genotyping revealed that the patient has the H1j/H2 diplotype, his mother H1j/H1j, his two healthy brothers H1j/H1v and his deceased father was by deduction H1v/H2. The H1j/H2 diplotype was shown in a total of 3 PD patients among 80, who also did not have known PD-causing mutation and in 1 out of 92 healthy individual controls. The three patients with this diplotype all have a similar clinical phenotype. Our results suggest that haplotypes H1j and H2 are strong risk factor alleles, and their combination could be responsible for early onset of PD with dementia.

## 1. Introduction

Parkinson's disease (PD) is a neurodegenerative condition that results in progressive movement disorder associated with nonmotor symptoms [1]. PD prevalence increases with age, approximately 1% of the individuals over age 60 years and 4% of the population older than age 85 [2]. The most typical PD motor features are bradykinesia, rest tremor, rigidity, and postural instability [3]. Also, PD patients can develop nonmotor symptoms such as sleep disturbance, fatigue, speech dysfunctions, depression, visual hallucinations, and dementia in 20 to 40% of cases [4]. Approximately, 5–10% of patients have monogenic forms of the disease with Mendelian dominant or recessive inheritance [5]. However, PD has a complex and multifactorial etiology that involves interactions between environmental and genetic factors [6]. Genome-wide association studies (GWAS) have linked PD to several susceptibility genes, among them microtubule-associated protein tau (MAPT) [7]. This gene expresses six different isoforms by alternative splicing depending on the presence or absence of exons 2, 3, and 10. The exon 10 splicing gives rise to three isoforms with 3 repeat microtubule-binding domain (3R) and three isoforms of 4 repeat microtubule-binding domain (4R) [8]. It is located on chromosome 17q21.3, in a region of complete linkage disequilibrium that covers ∼1.3–1.6 million bases (Mb), resulting in two distinct haplotypes H1 (direct orientation) and H2 (inverted orientation) due to a large inversion polymorphism ≈970 Kb, which are in complete disequilibrium and do not recombine [9]. The H1 haplotype is frequent in all populations, whereas the H2 haplotype is nearly absent in native Americans and East Asians, rare in Africans, and relatively more frequent (20–30%) in populations of Caucasian origin [10]. The H1 haplotype is heterogeneous compared with H2, and six SNPs are used to classify the different subclades (the rs9468 (C/T) to assign H1/H2 haplotype and five SNPs to define H1-specific subhaplotypes (rs1467967 (A/G), rs242557 (A/G), rs3785883 (A/G), rs2471738 (C/T), and rs7521 (A/G)) [11].

MAPT gene was identified as one of the strongest associated genes to PD and to a number of neurodegenerative diseases, such as Alzheimer's disease (AD), corticobasal degeneration (CBD), progressive supranuclear palsy (PSP), Parkinson's disease with dementia (PDD), and Parkinson's disease (PD) [12–14]. There are numerous studies that associate these diseases with the MAPT haplotypes H1 and H2. In fact, MAPT H1 haplotype has been found to be a risk factor for all these neurodegenerative disorders [15–17]. However, few and contradictory studies have been carried out on the association of haplotype H2 with neurodegenerative diseases risk. Indeed, some studies reported H2 haplotype as a protective haplotype in PD and AD [17–19], whereas others showed a significant association with AD, fronto-temporal dementia (FTD), and PD risks, related to a lower total MAPT expression [20–22].

In this work, we report a 48-year-old Moroccan patient displaying PD with dementia who had no known PD-causing mutation, but who carried a specific diplotype H1j/H2 of MAPT, suggesting that these two haplotypes are risk factor alleles, and their combination could be responsible for early onset PD with dementia.

## 2. Case Presentation

### 2.1. Clinical Data

All the studies were carried out after approval of the local ethical committee of biomedical research (CERB). All sampled family members provided informed consent to participate in the study.

**Figure 1:**
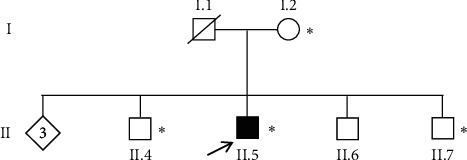
Pedigree of the family studied. ↗: index patient. ∗: genetic testing performed.

Patient II.5 is a 48-year-old man from an urban area in south Morocco, fifth of a sibling of 7 and born from a nonconsanguineous marriage (Figure 1). His father died at the age of 76 from natural causes, and his mother, still alive, has no medical history and is 75 years old. The patient presented at the age of 41 an akinesia of the right upper limb followed by the right lower limb that was responsive to dopaminergic drugs (ropinirole and levodopa). He had no tremor or gait impairment. Seven years later, he expressed motor fluctuations as delayed ON and wearing off phenomena. Entacapone was added to his treatment which improved motor fluctuations but set off mild dyskinesia. No axial symptoms were observed. As for nonmotor symptoms, the patient showed a progressive but mild cognitive decline 4 years after disease onset that evolved gradually later; the Mattis Dementia Rating Scale was 124/144 at the age of 48 years for a man with less than 5 years of education. He had no hallucination, but reported REM sleep behavior disorder (RBD) and chronic constipation. His dopamine equivalent daily dose was 1032 mg/day.

### 2.2. Genetics Results

Blood samples were taken from patient II.5 (reference 3595), his mother I.2, and two brothers II.4 and II.7. DNA was extracted using the Wizard® Genomic DNA Purification Kit (Promega Corporation). We first searched in the patient the most prevalent LRRK2 G2019S mutation in North African population, which was negative. Then, the multiplex ligation-dependent probe amplification (MLPA) was performed with the SALSA MLPA P051 Parkinson kit. Data analysis was performed using Coffalyser software (MRC, Holland, Amsterdam, The Netherlands), and results showed a normal copy number in all PD causal genes tested. After that, gene-panel next-generation sequencing (NGS) with 25 genes associated with PD and overlapping phenotypes (Supplementary Materials) (available here) was performed in the patient and his mother using the Ion Proton System for Next-Generation Sequencing (Thermo Fisher Scientific). The library was prepared by the Ion Chef System according to the Ion AmpliSeq Kit for Chef DL8 instructions. Bead templating (emulsion PCR) and chip loading were performed on Ion Chef System. Sequence alignment to Hg19 and variant identification was performed with the Torrent Suite v.4.2.1 software. The generated VCF was imported into the online Server of IonReporter Software v5.10 for variant analysis, filtering, and annotations. Results showed that the patient II.5 has 240 variants of which 14 were functional missense mutations (Table 1). These mutations have minor allele frequency above 0.1 in public databases, which were frequently found in inhouse individual healthy controls and were predicted to be benign. Nine of these 14 missense mutations were inherited from the mother and the other 5, all in exon 6 and 8 of the MAPT gene, come from the father and correspond to the H2 haplotype. Note that the mother has 101 SNVs including 17 functional missense mutations predicted all to be benign. Furthermore, the patient was heterozygous for 32 SNV in MAPT gene including exons, introns, 5′UTR, and 3′UTR corresponding to the diplotype H1/H2, whereas the mother has only 4 SNV and was homozygous for H1 haplotype.

MAPT subhaplotypes were then genotyped for all sampled family members using the six subhaplotypes tagging SNPs rs1467967 (A/G), rs242557 (A/G), rs3785883 (A/G), rs2471738 (C/T), rs9468 (T/C), and rs7521 (A/G) [11]. Results showed that mother I.2 was homozygous for H1j haplotype, patient II.5 was H1j/H2, whereas the two brothers II.3 and II.7 were H1j/H1v. The deceased father genotype was by deduction of H1v/H2. In order to confirm the implication of this H1j/H2 diplotype in PD causality, a cohort of 186 Moroccan PD patients, including 48 familial and 138 sporadic PD, and 92 healthy controls were genotyped for MAPT subhaplotypes. The mean age at onset was 50.85 ± 12.90 years old, and the mean disease duration was 5 ± 3.0 years, while the mean age at sampling of controls was 37.83 ± 11.19 years old. Genotyping results showed that the frequency of H1 haplotype reached 90.32% (336/372) of chromosomes in PD patients and 88.10% (162/184) in control chromosomes. The univariate logistic regression showed no significant difference in the frequency of the H1/H1 diplotype (Table 2) in cases compared to control subjects (81.7 vs. 77.2%; OR, 1.32; 95% CI, 0.62–2.44; *p* = 0.371). Interestingly, the H1j/H2 diplotype is absent in the 48 familial cases, present in 3 among the 138 sporadic cases and in only one among 92 control individuals who was of a young age of 37 years. In order to consider only cases with idiopathic PD patients without known genetic causes, like the case of the family studied here, we excluded from the cohort the familial forms which are often due to mutations in the Mendelian genes and the sporadic cases with the mutation LRRK2 G2019S which is the most common in North Africa. The 93 remaining sporadic cases were then analyzed by gene-panel NGS. Data analysis by the IonReporter Software showed that 80 PD patients among the 93 tested did not have pathogenic mutations in the known PD genes. The comparison of the frequencies of the H1j/H2 diplotype between sporadic idiopathic PD (3/80) and control subjects (1/92) showed a trend of association since the odds ratio was 3.35 which remains still not significant (3.75 vs. 1.0%; OR, 3.55; 95% CI, 0.36–34.78; *p* = 0.277) due to the rarity of the diplotype and the small size of the samples. It should be noted that two PD patients and one individual control homozygous for H2 haplotype were identical for all the 29 variants obtained in MAPT including SNVs and INDELs and define the H2 haplotype in Moroccan population (Supplementary Materials).

Clinical findings of the other two patients with the H1j/H2 diplotype discovered in our series of the 80 idiopathic PD patients presented a clinical phenotype similar to that of the case report 3598 (Table 3) in terms of age of onset, the initial symptom and the clinical form. Cognitive impairment was moderate in the patient 3894 with 3 years of disease evolution and absent in the patient 3793 but only with one year of disease evolution.

## 3. Discussion

We received in the Department of Neurology of Rabat a patient who was diagnosed with an akinetic-rigid form of PD and an early age of onset of 43 years old. The follow-up of the patient over a period of 7 years showed, in addition to motor fluctuations, a mild cognitive decline. Genetic analyzes by MLPA and gene-panel NGS performed in this patient did not show any pathogenic CNV or SNV in the known PD genes. However, in MAPT which is one of the strongest genetic risk factors for PD, he presented the H1j/H2 diplotype. This diplotype segregated in the family since it was absent in all the healthy family members tested including the mother and two brothers.

The major MAPT H1 haplotype has been associated with an increased risk of PD by several authors [14, 23, 24]. Since this haplotype is heterogeneous and was subdivided into 20 subhaplotypes [25], little is known, however, about which of these subhaplotypes could drive the association with PD. The rare subhaplotype MAPT H1j, with a frequency less than 2%, was reported to be significantly associated with PD risk [17, 26]. Recently, by genotyping a large series of brains of patients with Lewy body diseases including PD, PD with dementia, and dementia with Lewy body diseases, Heckman et al. [27] attributed this association to a direct association between H1j haplotype and a greater putaminal dopaminergic degeneration. These findings give very strong evidence to the implication of this rare H1j haplotype in the development of PD with dementia. However, the specific variant within this haplotype that determines the PD risk is not yet known. The MAPT H2 haplotype was reported to be associated with late-onset Alzheimer's disease risk related to a lower level of its brain gene expression [21]. It was also associated with global Parkinsonism in elderly specifically to its bradykinesia component, related to a lower MAPT expression particularly its specific isoform 1N/4R [22]. H2 haplotype occurred in 9.7% of our PD patient series and in 11.9% of control individuals, whereas it is rare in Africa, about 1%. The highest frequency is found in the European population, about 24% (1000 Genomes Project). The combination of these two risk haplotypes H1j and H2 was not found in any patient with familial PD, and the only 3 patients with this diplotype were all idiopathic sporadic patients without pathological mutation in the known PD genes, as was revealed by the gene-panel NGS analysis.

An additional argument in favor of the implication of this H1j/H2 diplotype in the PD risk was clinical since the clinical phenotype was similar in the three patients carrying this diplotype. Indeed, these patients started the disease at a young age between 43 and 47 years old, with akinesia as a symptom of onset and manifested progressively an akinetic-rigid form of Parkinsonism with good levodopa response. Index patient 3592 developed a slight cognitive impairment at the age of 45 that was objective 4 years later. Patient 3894 with 3 years of disease progression also showed a mild cognitive impairment, while patient 3793 with only one year of disease progression has not yet shown it. The only control individual with the H1j/H2 diplotype was 37 years of age and therefore has not yet reached the age of onset of the disease of the 3 patients with this diplotype. While each of these two haplotypes separately has been reported to be responsible for late-onset PD with bradykinesia and cognitive impairment, their combination into a diplotype could be responsible for early onset akinetic-rigid PD with cognitive impairment.

The discovery of this H1j/H2 diplotype in our patient was facilitated to the fact that the mother was homozygous for all the variants in the MAPT gene, which allowed to determine the H1j haplotype. The association of this diplotype with PD risk was subsequently searched in a series of 186 patients from which familial forms and isolated cases with a pathogenic mutation were excluded. One limitation of our study is the small sample size since only 80 idiopathic PD patients remained. However, most of the studies looking on the association of the MAPT subhaplotypes and the risk of developing neurodegenerative diseases have not analyzed all of the patients in their samples using NGS technologies. Thus, even if series were large, authors did not exclude from their sample patients who may have a pathogenic mutation in the known PD genes whether they are familial or sporadic cases. Furthermore, as the H1j haplotype is relatively rare, to reach statistical power, it will be necessary to conduct studies on large series of idiopathic PD patients from different ethnical origins that may be possible only within international collaborative approaches.

## Figures and Tables

**Table 1:** Functional mutations obtained by 25 gene-panel NGS in patient II.5.

Genes	Exon	Coding	Genotype	Amino acid change	Variant effect	dbSNP	MAF	Control chromosomes	CADD	ClinVar
DNAJC13	38	c.4387G > T	T/T	p.Ala1463Ser	Missense	rs3762672	0.273	25/40	18.78	NPC
EIF4G1	10	c.1315A > G	G/G	p.Met439Val	Missense	rs2178403	0.22	36/40	2.716	Benign
PARK2	10	c.1138G > C	C/G	p.Val380Leu	Missense	rs1801582	0.144	05/40	2.420	Benign
LRRK2	49	c.7190T > C	T/C	p.Met2397Thr	Missense	rs3761863	0.45	24/40	1.618	Benign
VPS13C	64	c.8738G > A	C/T	p.Ser2913Asn	Missense	rs10851704	0.461	20/40	19.03	NPC
VPS13C	29	c.2921G > A	C/T	p.Arg974Lys	Missense	rs3784634	0.282	27/40	16.27	NPC
POLG	23	c.3708G > T	C/A	p.Gln1236His	Missense	rs3087374	0.037	06/40	21.4	Benign
MAPT	6	c.605C > T	C/T	p.Pro202Leu	Missense	rs63750417	0.117	02/40	13.58	Benign
MAPT	6	c.853G > A	G/A	p.Asp285Asn	Missense	rs62063786	0.117	02/40	8.095	Benign
MAPT	6	c.866T > C	T/C	p.Val289Ala	Missense	rs62063787	0.117	02/40	0.539	Benign
MAPT	6	c.1108C > T	C/T	p.Arg370Trp	Missense	rs17651549	0.116	02/40	24.4	Benign
MAPT	8	c.1321T > C	T/C	p.Tyr441His	Missense	rs2258689	0.324	18/40	15.38	Benign
MAPT	8	c.1339T > C	T/C	p.Ser447Pro	Missense	rs10445337	0.117	02/40	18.32	Benign
SYNJ1	8	c.1001A > G	T/C	p.Lys334Arg	Missense	rs2254562	0.292	11/40	27.1	NPC

NPC: not provided in ClinVar.

**Table 2:** Comparison of frequencies of the MAPT diplotypes between PD patients and controls.

Subjects (N)	Diplotype, *n* (%)			OR	*p* value
	H1/H1	H1/H2 or H2/H2	H1j/H2		
All PD (186)	152 (81,7)	34 (18,3)	3 (1,61)		
Familial PD (48)	41 (85,4)	7 (14,6)	0 (0,00)		
Sporadic PD (138)	111 (80,4)	27 (19,6)	3 (2,17)		
Idiopathic sporadic PD (80)	63 (78,7)	14 (17,2)	3 (3,75)	3,55	0,277
Control subjects (92)	71 (77,2)	21 (22,8)	1 (1,08)		

**Table 3:** Clinical features of the 3 Moroccan PD patients with the MAPT H2/H1j genotype.

Patients	3592	3793	3894
Sex	M	M	F
Consanguinity	−	−	−
Age at onset	41	47	47
Disease duration	7 years	1 year	3 years
Initial symptom	Akinesia	Akinesia	Akinesia
Clinical form	Akinetic-rigid	Akinetic-rigid	Mixed
Resting tremor	−	−	+
Akinesia	+	+	+
Rigidity	+	+	+
Dystonia	−	−	−
Gait impairment	−	+	+
Postural instability	−	−	−
UPDRS III (on)	11	10	18
H–Y score	1	1.5	2
Motor fluctuation	+	−	+
Levodopa induced dyskinesia	+	−	−
Levodopa equivalent dose	1032	400	600
Urinary dysfunction	−	−	−
Orthostatic HypoTA	−	−	+
Pain	−	+	+
Constipation	+	−	+++
Sleep disorder	+	−	+
Psychiatric features	−	−	−
Cognitive decline	+	−	+

## Data Availability

The data used to support the findings of this study are included within the article and the supplementary information file.

## Supplementary Materials

Supplementary Materials The Supplementary Material contains the NGS gene panel, all the mutations identified in the Moroccan MAPT H2 haplotype and H1j haplotype, and the epidemiological data of Idiopathic PD and control individuals.
