# Supplementary material for: A Specific Diplotype H1j/H2 of the MAPT Gene Could Be Responsible for Parkinson's Disease with Dementia
Source: Case Rep Genet. 2020 Dec 3;2020:8813344. doi: 10.1155/2020/8813344 (PMC7732378; doi:10.1155/2020/8813344)
Supplement: Supplementary Materials — The Supplementary Material contains the NGS gene panel, all the mutations identified in the Moroccan MAPT H2 haplotype and H1j haplotype, and the epidemiological data of Idiopathic PD and control individuals. [file 8813344.f1.zip › 8813344.f1/Supplementary Material 02.pdf]

Variants of the MAPT H2/H2 diplotype

Locus	Genotyp	Ref	Observ	Type	Genes	% Frequer	Exon
chr17:44039691	G/G	A	G	SNV	**MAPT**	100.00	2
chr17:44060775	T/T	C	T	SNV	**MAPT**	99.55	6
chr17:44061023	A/A	G	A	SNV	**MAPT**	99.87	6
chr17:44061036	C/C	T	C	SNV	**MAPT**	99.82	6
chr17:44061278	T/T	C	T	SNV	**MAPT**	95.28	6
chr17:44067400	C/C	T	C	SNV	**MAPT**	93.39	8
chr17:44068924	A/A	G	A	SNV	**MAPT**	99.93	9
chr17:44071294	C/C	T	C	SNV	**MAPT**	100.00	10
chr17:44073889	G/G	A	G	SNV	**MAPT**	99.92	11
chr17:44073973	C/C	T	C	SNV	**MAPT**	100.00	11
chr17:44101563	C/C	T	C	SNV	**MAPT**	99.87	15
chr17:44101871	G/G	A	G	SNV	**MAPT**	100.00	15
chr17:44102443	CCT/CCT	C	CCT	INDEL	**MAPT**	98.56	15
chr17:44102604	C/C	T	C	SNV	**MAPT**	99.83	15
chr17:44102638	G/G	A	G	SNV	**MAPT**	99.97	15
chr17:44102682	CT/CT	C	CT,T	INDEL	**MAPT**	:99.35, T=0	15
chr17:44102689	C/C	G	C	SNV	**MAPT**	100.00	15
chr17:44102741	T/T	TCA	T	INDEL	**MAPT**	100.00	15
chr17:44102865	C/C	A	C	SNV	**MAPT**	99.52	15
chr17:44102933	C/C	T	C	SNV	**MAPT**	100.00	15
chr17:44103296	C/C	T	C	SNV	**MAPT**	97.51	15
chr17:44103616	T/T	C	T	SNV	**MAPT**	99.85	15
chr17:44103825	CA/CA	TG	CA	MNV	**MAPT**	99.97	15
chr17:44104278	T/T	TG	T	INDEL	**MAPT**	100.00	15
chr17:44104343	C/C	A	C	SNV	**MAPT**	99.85	15
chr17:44104411	C/C	CTCC	C	INDEL	**MAPT**	100.00	15
chr17:44104509	C/C	T	C	SNV	**MAPT**	99.97	15
chr17:44104576	C/C	CCTT	C	INDEL	**MAPT**	99.95	15
chr17:44105395	G/G	A	G	SNV	**MAPT**	99.90	15

Coding	Amino Acid Cha	Variant Effect	dbSNP	ClinVar	Coverage	MAF
c.-13A>G	**p.?**	unknown	**rs17650901**	Benign	254	0.086
c.605C>T	**p.Pro202Leu**	missense	**rs63750417**	Benign	3996	0.088
c.853G>A	**p.Asp285Asn**	missense	**rs62063786**	Benign	3996	0.088
c.866T>C	**p.Val289Ala**	missense	**rs62063787**	Benign	3965	0.088
c.1108C>T	**p.Arg370Trp**	missense	**rs17651549**	Benign	1018	0.086
c.1339T>C	**p.Ser447Pro**	missense	**rs10445337**	Benign	2223	0.088
c.1479G>A	**p.(=)**	synonymous	**rs1052551**	Benign	1340	0.086
c.1512T>C	**p.(=)**	synonymous	**rs62063845**	Benign	3993	0.088
c.1686A>G	**p.(=)**	synonymous	**rs1052553**	Benign	3993	0.088
c.1770T>C	**p.(=)**	synonymous	**rs17652121**	Benign	3999	0.088
c.2357T>C	**p.?**	unknown	**rs9468**	Benign	3971	0.086
c.2665A>G	**p.?**	unknown	**rs8712**	Benign	1259	0.086
c.3237_3238insCT	**p.?**	unknown	**rs113598111,rs!**	Benign	139	0.086
c.3398T>C	**p.?**	unknown	**rs1052587**	Benign	2888	0.086
c.3432A>G	**p.?**	unknown	**rs1052590**	Benign	3983	0.086
c.3476_3477insT	**p.?**	unknown	**rs5820605**	Benign	3989	0.382
c.3483G>C	**p.?**	unknown	**rs1052594**	Benign	3994	0.086
c.3536_3537delCA	**p.?**	unknown		Benign	3978	
c.3659A>C	**p.?**	unknown	**rs17574040**	Benign	3993	0.086
c.3727T>C	**p.?**	unknown	**rs16940799**	Benign	3995	0.086
c.4090T>C	**p.?**	unknown	**rs7687**	Benign	3818	0.086
c.4410C>T	**p.?**	unknown	**rs17652748**	Benign	3992	0.086
c.4619_4620delTGinsCA	**p.?**	unknown	**rs16940806,rs7!**	VUS	3984	0.086
c.5073delG	**p.?**	unknown	**rs5820606**	Benign	3671	0.086
c.5137A>C	**p.?**	unknown	**rs2158257**	Benign	3320	0.086
c.5206_5208delTCC	**p.?**	unknown	**rs199706121,rs56847546(**		2364	0.086
c.5303T>C	**p.?**	unknown	**rs17574228**	Benign	3985	0.086
c.5371_5373delCTT	**p.?**	unknown	**rs760422692**	Benign	3972	0.174
c.6189A>G	**p.?**	unknown	**rs7521**	Benign	3999	0.386 (ı

0.386 (ref)
