# Supplementary material for: A Specific Diplotype H1j/H2 of the MAPT Gene Could Be Responsible for Parkinson's Disease with Dementia
Source: Case Rep Genet. 2020 Dec 3;2020:8813344. doi: 10.1155/2020/8813344 (PMC7732378; doi:10.1155/2020/8813344)
Supplement: Supplementary Materials — The Supplementary Material contains the NGS gene panel, all the mutations identified in the Moroccan MAPT H2 haplotype and H1j haplotype, and the epidemiological data of Idiopathic PD and control individuals. [file 8813344.f1.zip › 8813344.f1/Supplementary Material 03.pdf]

Variants of the H1J/H1J diplotype

Locus	Genotype	Ref	Observe	Type	Genes	Length	% Frequer	Exon	Transcript	Coding	Amino Acid Char
chr17:44061025	T/T	C	T	SNV	**MAPT**	1	99.77	6	NM_001123(	c.855C>T	**p.(=)**
chr17:44067382	C/C	T	C	SNV	**MAPT**	1	100.00	8	NM_001123(	c.1321T>C	**p.Tyr441His**
chr17:44104612	TG/TG	T	TG	INDEL	**MAPT**	1	100.00	15	NM_001123(	c.5406_5407insG	**p.?**
chr17:44105395	G/G	A	G	SNV	**MAPT**	1	99.98	15	NM_001123(	c.6189A>G	**p.?**

Variant Effect	dbSNP	ClinVar	Coverage	MAF
synonymous	rs63750222	Benign	3998	0.11
missense	rs2258689	Benign	1676	0.313
unknown	rs151340107,rs	Benign	3972	0.205
unknown	rs7521	Benign	3996	0.386 (ref)
