# Supplementary material for: A Specific Diplotype H1j/H2 of the MAPT Gene Could Be Responsible for Parkinson's Disease with Dementia
Source: Case Rep Genet. 2020 Dec 3;2020:8813344. doi: 10.1155/2020/8813344 (PMC7732378; doi:10.1155/2020/8813344)
Supplement: Supplementary Materials — The Supplementary Material contains the NGS gene panel, all the mutations identified in the Moroccan MAPT H2 haplotype and H1j haplotype, and the epidemiological data of Idiopathic PD and control individuals. [file 8813344.f1.zip › 8813344.f1/Supplementary Material 04.pdf]

Epidemiological data of Idiopathic PD and individual controls

Individual ID	Gender	Age	Age at onset	Ethnic origin
**3159**	F	60	57	Arabic
**3214**	F	66	50	Arabic
**3216**	F	64	58	Berber
**3228**	H	70	52	Arabic
**3316**	H	58	48	Berber
**3317**	H	49	44	Berber
**3334**	H	67	60	Arabe
**3348**	F	69	65	Arabic
**3370**	H	66	62	Arabic
**3435**	H	51	45	Arabic
**3507**	H	62	58	Arabic
**3508**	H	54	53	Mixed
**3533**	H	37	28	Mixed
**3536**	F	46	42	Arabic
**3537**	F	62	52	Berber
**3573**	H	62	52	Berber
**3588**	F	62	55	Arabic
**3592**	H	43	41	Berber
**3593**	H	80	73	Berber
**3600**	H	69	64	Arabic
**3623**	H	57	34	Arabic
**3626**	F	62	60	Arabic
**3632**	F	61	59	Mixed
**3650**	F	68	62	Mixed
**3666**	H	62	61	Arabic
**3671**	H	80	64	Berber
**3695**	F	55	53	Arabic
**3710**	H	47	44	Arabic
**3711**	H	53	47	Arabic
**3754**	F	65	60	Arabic
**3761**	H	51	49	Arabic
**3779**	F	60	50	Mixed
**3786**	H	64	62	Arabic
**3793**	H	48	46	Arabic
**3794**	H	60	54	Berber
**3797**	F	65	60	Berber
**3798**	H	66	53	Arabic
**3801**	F	60	59	Arabic
**3802**	F	75	70	Arabic
**3808**	F	75	55	Arabic
**3812**	H	57	45	Arabic
**3814**	H	68	60	Arabic
**3815**	H	70	66	Arabic
**3818**	F	45	37	Mixed
**3821**	H	49	44	Arabic
**3822**	H	65	55	Arabic
**3824**	H	68	64	Arabic
**3847**	H	71	69	Berber
**3851**	H	45	37	Berber

**3852**	F	80	76	Arabic
**3853**	H	63	57	Arabic
**3854**	F	54	49	Arabic
**3855**	H	30	27	Arabic
**3856**	F	53	50	Arabic
**3860**	H	52	40	Berber
**3861**	F	58	46	Berber
**3864**	H	67	65	Arabic
**3870**	H	64	48	Arabic
**3871**	H	66	45	Mixed
**3876**	H	58	54	Arabic
**3877**	H	65	64	Arabic
**3880**	H	63	63	Arabic
**3890**	F	53	46	Arabic
**3892**	F	57	35	Arabic
**3893**	M	70	68	Mixed
**3894**	F	59	57	Berber
**3895**	F	49	47	Arabic
**3898**	M	53	51	Berber
**3900**	M	76	60	Arabe
**3901**	M	43	37	Berber
**3902**	M	63	52	Arabic
**3907**	H	44	38	Arabic
**3908**	H	62	58	Arabic
**3909**	F	62	60	Arabic
**3913**	H	60	60	Berber
**3928**	H	35	31	Arabic
**3931**	F	55	42	Arabic
**3941**	F	54	49	Arabic
**3947**	H	76	72	Arabic
**3957**	F	65	65	Arabic
**C002**	F	37		Arabic
**C007**	M	25		Arabic
**C008**	F	24		Berber
**C009**	M	52		Arabic
**C010**	M	52		Arabic
**C011**	M	27		Arabic
**C012**	M	28		Berber
**C013**	M	43		Berber
**C016**	H	30		Mixed
**C017**	F	31		Arabic
**C018**	M	26		Arabic
**C019**	M	34		Arabic
**C020**	M	33		Berber
**C021**	M	24		Berber
**C025**	M	43		Arabic
**C030**	M	39		Arabic
**C032**	M	36		Arabic
**C034**	M	24		Arabic
**C037**	M	31		Arabic
**C038**	M	36		Mixed
**C039**	M	54		Mixed

C041	M	37		Mixed
C044	M	32		Berber
C045	M	39		Mixed
C046	M	33		Arabic
C047	F	40		Arabic
C049	F	34		Mixed
C050	F	35		Arabic
C052	F	37		Berber
C054	F	28		Arabic
C055	M	29		Berber
C057	M	43		Arabic
C058	M	25		Arabic
C060	M	47		Arabic
C061	M	29		Arabic
C062	F	41		Arabic
C066	M	40		Berber
C067	M	28		Arabic
C068	M	26		Arabic
C069	M	36		Arabic
C070	F	45		Berber
C071	F	40		Arabic
C072	F	20		Mixed
C073	F	21		Arabic
C075	F	22		Arabic
C076	M	37		Mixed
C077	M	51		Berber
C079	M	58		Arabic
C080	F	47		Mixed
C082	M	57		Mixed
C083	F	24		Berber
C084	M	35		Arabic
C085	M	54		Arabic
C086	M	41		Berber
C088	F	20		Arabic
C090	F	31		Arabic
C091	M	28		Berber
C094	F	43		Arabic
C095	F	31		Arabic
C096	F	58		Arabic
C097	M	36		Arabic
C098	F	20		Arabic
C099	F	30		Mixed
C100	F	59		Berber
C101	F	46		Arabic
C102	F	43		Arabic
C103	F	26		Berber
C104	F	56		Mixed
C106	M	46		Arabic
C108	M	50		Berber
C110	F	21		Arabic
C111	M	32		Berber
C113	F	41		Berber

**C117**	M	59		Arabic
**C118**	M	36		Berber
**C121**	F	44		Berber
**C122**	F	23		Arabic
**C123**	F	44		Berber
**C124**	F	44		Arabic
**C130**	M	33		Berber
**C137**	M	44		Berber
**C160**	M	34		Mixed
**C163**	F	44		Arabic
**C164**	F	33		Mixed
**C165**	M	33		Arabic
**C171**	M	60		Arabic
**C177**	M	39		Mixed
**C180**	M	54		Berber
**C193**	F	23		Mixed
**C196**	M	60		Arabic
**C198**	F	60		Mixed
**C201**	M	56		Arabic
