# Supplementary material for: A Specific Diplotype H1j/H2 of the MAPT Gene Could Be Responsible for Parkinson's Disease with Dementia
Source: Case Rep Genet. 2020 Dec 3;2020:8813344. doi: 10.1155/2020/8813344 (PMC7732378; doi:10.1155/2020/8813344)
Supplement: Supplementary Materials — The Supplementary Material contains the NGS gene panel, all the mutations identified in the Moroccan MAPT H2 haplotype and H1j haplotype, and the epidemiological data of Idiopathic PD and control individuals. [file 8813344.f1.zip › 8813344.f1/Supplementary Material O1.pdf]

List of genes of the NGS panel used

Gene	Disease	Location	Transmi	OMIM
**SNCA**	Parkinson disease 1, 4; Dementia, Lewy body	4q22.1	AD	https://omim.org/entry/163890
**LRRK2**	Parkinson disease 8	12q12	AD	https://omim.org/entry/609007
**VPS35**	Parkinson disease 17	16q11.2	AD	https://omim.org/entry/601501
**PARK2**	Parkinson disease 2	6q26	AR	https://omim.org/entry/600116
**PARK7**	Parkinson disease 7	1p36.23	AR	https://omim.org/entry/602533
**PINK1**	Parkinson disease 6	1p36.12	AR	https://omim.org/entry/608309
**PLA2G6**	Parkinson disease 14	22q13.1	AR	https://omim.org/entry/603604
**VPS13C**	Parkinson disease 23	15q22.2	AR	https://omim.org/entry/608879
**SYNJ1**	Parkinson disease 20	21q22.11	AR	https://omim.org/entry/604297
**ATP13A2**	Kufor-Rakeb syndrome	1p36.13	AR	https://omim.org/entry/610513
**DNAJC6**	Parkinson disease 19	1p31.3	AR	https://omim.org/entry/608375
**FBXO7**	Parkinson disease 15	22q12.3	AR	https://omim.org/entry/605648
**GCH1**	Dystonia, DOPA-responsive	14q22.2	AD, AR	https://omim.org/entry/600225
**GBA**	Gaucher disease	1q22	AR	https://omim.org/entry/606463
**POLG**	Mitochondrial DNA depletion syndrome	15q26.1	AR	https://omim.org/entry/174763
**ATP6AP2**	Parkinsonism with spasticity	Xp11.4	XLR	https://omim.org/entry/300556
**PANK2**	Neurodegeneration with brain iron accumulation 1	20p13	AR	https://omim.org/entry/606157
**MAPT**	Parkinson disease, susceptibility to	17q21.31	AD	https://omim.org/entry/157140
**EIF4G1**	Parkinson disease, susceptibility to	3q27.1	AD	https://omim.org/entry/600495
**UCHL1**	Parkinson disease, susceptibility to	4p.13	AD	https://omim.org/entry/191342
**GIGYF2**	Parkinson disease, susceptibility to	2q37.1	AD	https://omim.org/entry/612003
**DNAJC13**	Parkinson disease, susceptibility to	3q22.1	AD	https://omim.org/entry/614334
**HTRA2**	Parkinson disease, susceptibility to	p13.1	AD	https://omim.org/entry/606441
**GAK**	Parkinson disease, susceptibility to	4p16.3	?	https://omim.org/entry/602052
**RAB7L1**	Parkinson disease, susceptibility to	1q32.1	?	https://omim.org/entry/603949
